# Prospective study of clinician-entered research data in the Emergency Department using an Internet-based system after the HIPAA Privacy Rule

**DOI:** 10.1186/1472-6947-4-17

**Published:** 2004-10-12

**Authors:** Jeffrey A Kline, Charles L Johnson, William B Webb, Michael S Runyon

**Affiliations:** 1 Department of Emergency Medicine, Carolinas Medical Center, Charlotte, NC, USA; 2 BreathQuant Medical Systems Inc, Charlotte NC, USA

## Abstract

**Background:**

Design and test the reliability of a web-based system for multicenter, real-time collection of data in the emergency department (ED), under waiver of authorization, in compliance with HIPAA.

**Methods:**

This was a phase I, two-hospital study of patients undergoing evaluation for possible pulmonary embolism. Data were collected by on-duty clinicians on an HTML data collection form (prospective e-form), populated using either a personal digital assistant (PDA) or personal computer (PC). Data forms were uploaded to a central, offsite server using secure socket protocol transfer. Each form was assigned a unique identifier, and all PHI data were encrypted, but were password-accessible by authorized research personnel to complete a follow-up e-form.

**Results:**

From April 15, 2003-April 15 2004, 1022 prospective e-forms and 605 follow-up e-forms were uploaded. Complexities of PDA use compelled clinicians to use PCs in the ED for data entry for most forms. No data were lost and server log query revealed no unauthorized entry. Prospectively obtained PHI data, encrypted upon server upload, were successfully decrypted using password-protected access to allow follow-up without difficulty in 605 cases. Non-PHI data from prospective and follow-up forms were available to the study investigators via standard file transfer protocol.

**Conclusions:**

Data can be accurately collected from on-duty clinicians in the ED using real-time, PC-Internet data entry in compliance with the Privacy Rule. Deidentification-reidentification of PHI was successfully accomplished by a password-protected encryption-deencryption mechanism to permit follow-up by approved research personnel.

## Background

The ability of medical researchers to obtain and store electronic clinical data was complicated by requirements of the Patient Privacy Rule of the Health Insurance Portability and Accountability Act ("HIPAA") of 1996 in title 45 of the Federal Register, parts 160, subparts A and E of part 164 [1-3]. The HIPAA creates a conflict for investigators. The law specifies that 18 data elements, known as protected health identifiers (PHI), that could be used to identify the patient, must be adequately protected from disclosure. However, to allow follow-up, the investigator usually must collect PHI. In the most conservative interpretation of the Privacy Rule, investigators must obtain written informed consent and written authorization to collect PHI. In the hectic setting of the emergency department, the step of obtaining written authorization can bias the data sample [4]. The Privacy Rule does allow PHI to be collected without written authorization if the institutional Privacy Board grants waiver of authorization. Waiver of authorization requires special handling of PHI.

Existing electronic data collection methods are limited in their ability to centralize data in a fashion that expedites data sharing while remaining in compliance with HIPAA. For example, commercial spreadsheets that run on Windows^® ^do not mandate user identification, do not partition and encrypt sensitive data, and do not maintain a record and audit trail of use. Accordingly, we developed a comprehensive electronic system for clinicians to capture clinical research data from the bedside using commercially available hardware and data upload over the Internet. The system was programmed with multiple security steps and authentication procedures to maintain data security and privacy.

We tested the hypothesis that real-time clinical data can be obtained from clinicians in multiple hospitals using electronic data collection stored in an off-site server, under waiver of Authorization, while remaining in compliance with the Privacy Rule. This study represents the development and implementation phase of an ongoing multicenter study to collect prospective and follow-up clinical data from patients undergoing evaluation for pulmonary embolism in the emergency department. The specific aims of this study were to: 1. Test the feasibility of real-time, electronic data collection on personal digital assistants and personal computers in the emergency department setting in two hospitals. 2. Test if the system would correctly upload protected health information (PHI) in a secure and encrypted fashion, but allow follow-up to be performed by selected individuals using password-protected access to PHI.

## Methods

### Human subjects and Institutional approval

Patients were enrolled from two hospitals in Charlotte, NC: Carolinas Medical Center Main and Carolinas Medical Center University. The clinical protocol was approved under waiver of informed consent and waiver of authorization by the Carolinas HealthCare Institutional Review Board and the Institutional Privacy Board in accordance with the published guidelines of the Department of Health and Human Services (DHHS) [5], which were reviewed by Annas [6].

Because of institutional sensitivity about maintaining compliance with the privacy rule, this project required intensive planning and due diligence. Over a 6-month period, the authors scheduled several meetings with the Director of Privacy in Clinical Research, the hospital's Assistant Vice President of Privacy, and the Director of Information Security to discuss the protocol and methods. These individuals had oversight for privacy issues for both hospitals. Then, to facilitate the process of gaining assistance and approval from the Information Systems Department in implementing the technical aspects (software deployment and firewall access) at both hospitals, we obtained a letter of approval from each of these individuals to physically show to technical support personnel.

All patients in this study underwent evaluation for pulmonary embolism. The method of selection and diagnosis have been described previously [7]. For each patient, two electronic data forms ("e-forms") were (or will be) completed. The first was a prospective e-form that was completed in real-time in the emergency department by the clinician in charge of the patient's care. The second e-form encoded follow-up data, and was completed 45 days or more after the prospective form. The follow-up e-form was completed by one of two research associates. This study was non-interventional.

### System overview

**Figure 1:**
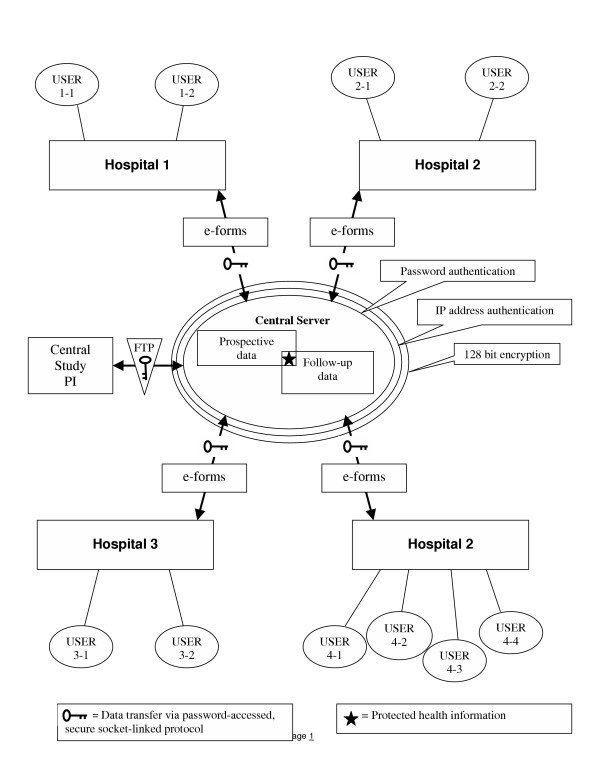
Schematic of system structure.

This system was designed to allow data to be transferred from multiple sites and stored on one server using technical requirements described in part 160 and 164 of the Privacy Rule. Figure 1 shows a schematic of the overall system structure with the hypothetical participation of four sites. According to published recommendations of the DHHS, the overarching requirement for collection of databases under waiver of authorization is to ensure de-identification of data. The DHHS specifies that this can be done either by the "safe harbor" approach, which entails removal, or the "statistical probability" method, which for practical purposes, incorporates data encryption/de-encryption techniques. The present system uses the statistical probability method, whereby the PHI data are subjected to 128-key bit encryption prior to upload on the server, but are linked to a non-PHI unique identifier (e.g., CMC0001) that allows joining of non-PHI data with PHI data for the purpose of conducting follow-up (see the star in the middle of the schematic in Figure 1). This step allows a research coordinator with the appropriate login and password to access de-encrypted (re-identified) data. In the final step, an FTP protocol was used to download the non-PHI follow-up data together with the correct prospective data for each patient. Both the prospective and follow-up data were exported in table form, one row per patient. In summary, authorized research personnel from each site had password-protected access to PHI of patients from their hospital only, while unauthorized personnel could access non-PHI study data via an FTP. An example of the latter would occur in Figure 1 if the site PI from hospital 1 were interested in viewing research data collected at hospital 3. The description of the individual elements of the system that follows is presented in the order that the study was conducted.

### Data entry form structure

**Figure 2:**
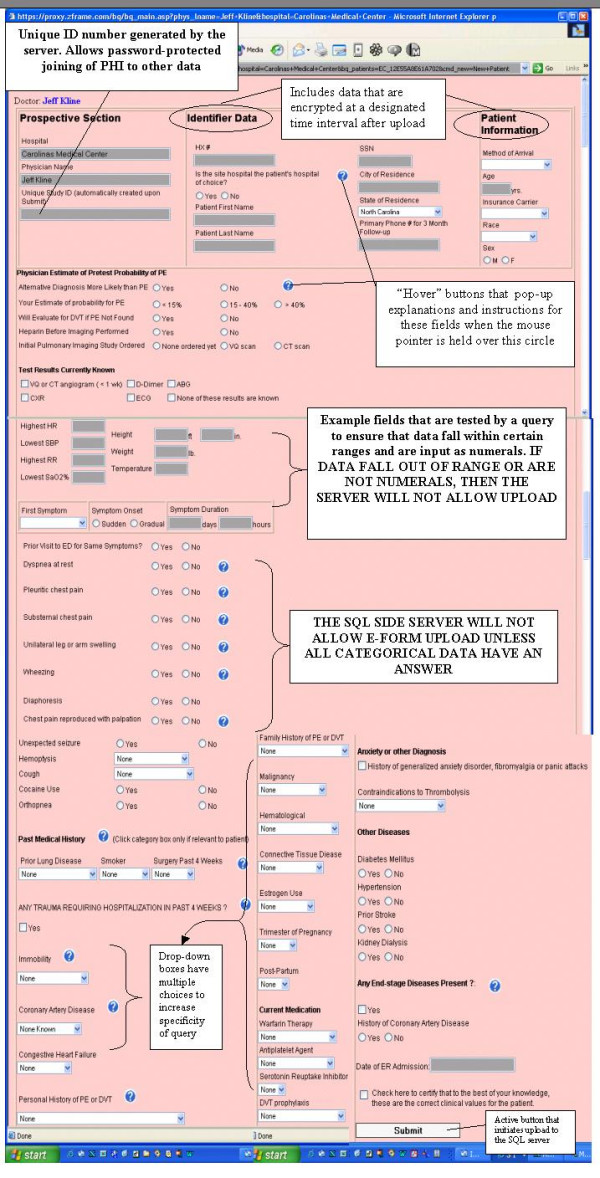
Reproduction of the prospective e-form as seen by the user.

**Figure 3:**
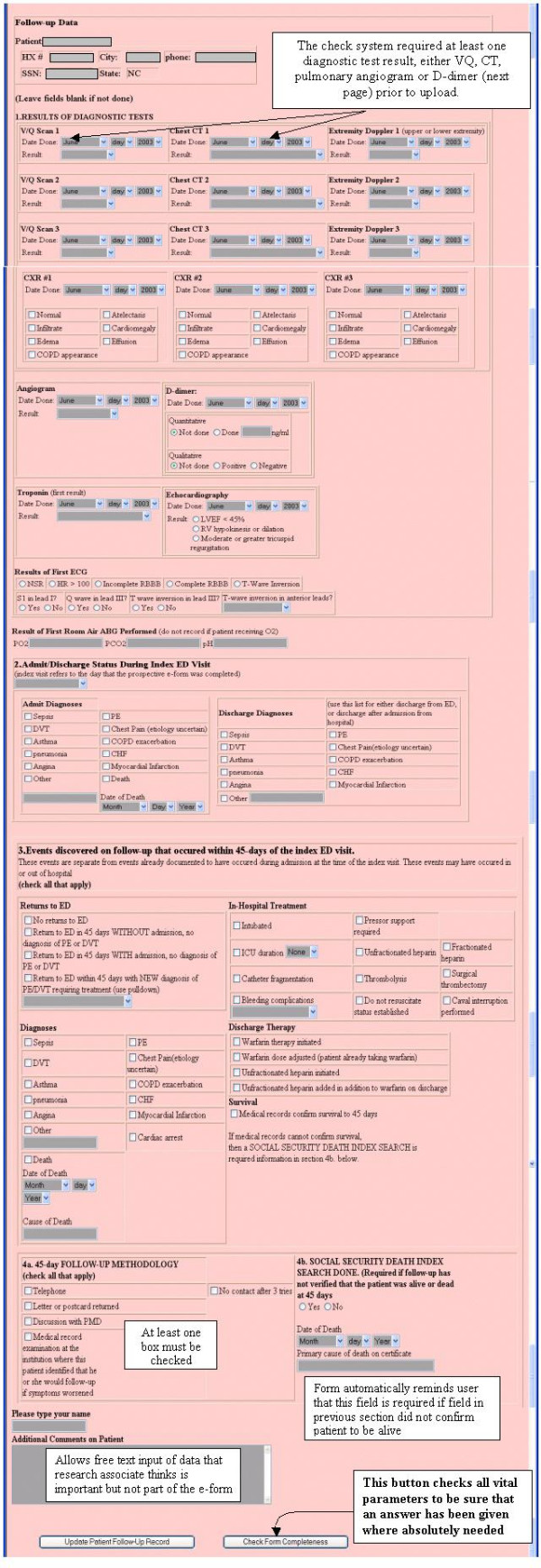
Reproduction of the follow-up e-form as seen by the user.

The trigger for data entry was the decision to order a diagnostic test to rule out pulmonary embolism in a symptomatic emergency department patient. Patient data were entered on the prospective e-form. The e-forms were programmed using hypertext markup language (HTML) in conjunction with active server pages (ASP) and Standard Query Language (SQL). The prospective and follow-up e-forms are shown in Figures 2 and 3. The prospective e-form contained a total of 70 fields for data entry including text strings, pull-downs, and click portals. The explicit definition of each field was provided by embedded text that could be viewed by mouse click over an adjacent question mark. These terms are defined in the Table 1. When the user executed an e-form upload, the server-side ASP code queried data fields for presence of an entry and validity of the entry. For parametric data, such as heart rate, the side-code query interrogated whether numerals had been entered and whether the number fell within a defined range. For example, the heart rate entry had to fall within 21 and 200 beats per minute. (If the investigator encountered a patient with a parameter outside of the allowed range, he or she could click an email link to notify the study administrator, who could override the system to make the entry.) Likewise, if the form contained a missing field, or a nonsensical entry from keystroke error (e.g. a heart rate of "t3"), the server would not load the form, and an error message directed the clinician to the field requiring correction and highlighted the erroneous entry in red shading. Once the field was corrected, the form could be uploaded.

To test for data validity in the prospective forms uploaded by clinicians, two authors independently examined each of 70 fields for all patients uploaded. We evaluated for blank cells, nonsensical character entry, or numeric entry that fell outside the predefined ranges.

### Real-time data entry

Forms were completed by attending physicians (N = 22), resident physicians (N = 20), and physician assistants (N = 6) in two emergency departments while the patient of interest was still in the emergency department. Prior to study deployment, each clinician was individually trained in a 10-minute session by the study principal investigator using a pre-defined protocol, and each clinician received a follow-up letter that summarized the training session. Data forms could be accessed in the emergency department using designated Internet-connected personal computers within patient care areas, or could be completed using individually owned personal digital assistants using the Pocket PC^® ^operating system followed by synchronized upload to the hosted server. All clinicians owned a compatible PDA. The authors and staff assistants provided technical support to assist clinicians in the process of downloading the prospective e-form to their PDAs and uploading completed e-forms to the study's central server using commercial software (Microsoft ActiveSynch^® ^v. 3.5). The clinicians were shown that prospective data entry forms could also be accessed through a URL hyperlink that was posted on the desktop of all Internet-connected computers in both emergency departments. When the user clicked the hyperlink, this action routed the user through the firewall directly to the hosted server for this study. All computers ran Windows 95 or higher, with ethernet connection to a T3 44.736 Mbps channel.

**Figure 4:**
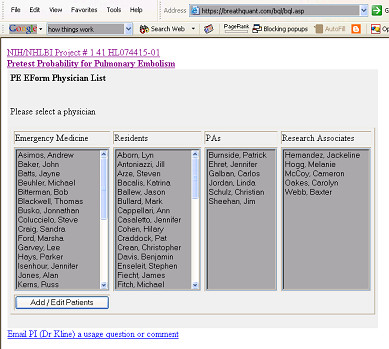
Login web page for clinicians to access prospective e-forms.

Upon opening the first web page, the user viewed a list of clinician names (Figure 4). The clinician then chose his or her name and opened a new, blank prospective e-form. No login was required to access or upload the form, but the central server was programmed to accept uploaded e-forms only from Internet provider addresses of the computers in the two emergency departments. When each new, complete prospective e-form was uploaded to the hosted server, the server encoded the e-form with a unique identification number bearing the initials of the hospital where data originated, the sequence number and clinician who entered the data. (e.g., CMC023JAK).

### Privacy controls

Multiple methods were used to ensure that protected health information would not be subject to unauthorized access, viewing or hijacking. When clinicians entered data in the emergency department, the server polled the form for inactivity exceeding 30 minutes, at which time the page would automatically close without being uploaded. We anticipated scenarios where a clinician would enter data on a prospective form that would need to be revised as a result of updated information (e.g., access to additional medical records, or arrival of family). To allow for such editing, the clinician could re-access any prospective form for a period of 60 minutes after initial upload, provided that the Internet provider address of the computer was the same as the computer from which the form originated. After 60 minutes, the prospective e-form could be altered only by a study administrator. All data were transferred using secure socket link (SSL) protocol. The central server (Win2000 OS) was located off-site at a large commercial web hosting facility (NTT/Verio Inc). Upon upload, all fields containing any of the 18 elements of that constitute PHI were subjected to 128 key-bit encryption. Data were stored in relational tables. To allow data analysis for research purposes, the study PI could access stored by file transfer protocol and exported into a format compatible with commercial software (e.g., Microsoft Access^®^, Seattle WA). However, all PHI data fields were remained encrypted.

### Follow-up data entry

**Figure 5:**
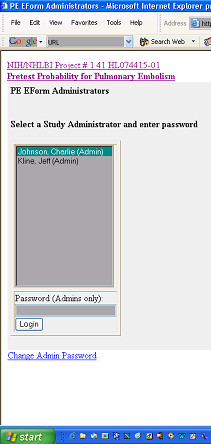
Login web page to allow secure authenticated access to follow-up e-forms.

**Figure 6:**
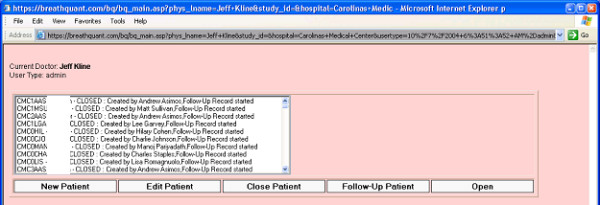
User view after successful authentication. From this list, the desired follow-up e-forms can be opened and populated.

Patients were then followed prospectively to determine outcome at 45 days. The follow-up data were entered by an IRB and privacy board approved, designated research coordinator. Because follow-up mandated access to PHI data, a separate web page was developed to allow the study coordinator to have administrative access to the necessary data. The research coordinator would type the appropriate URL address and then view a login page (Figure 5). The research coordinator could obtain password-protected access to the list of all uploaded prospective data forms (Figure 6), and upon mouse click of the "Follow-up Patient" button, the follow-up form was displayed with the required data to assist in follow-up, including patient name, medical record number, social security number, and telephone number (see top of Figure 2). This system thus allowed upload of prospective and follow-up e-forms from multiple hospitals, while research coordinators with IRB and privacy board approval could view the minimum PHI required to perform follow-up at their hospital. Research coordinators could not view PHI from other hospitals. However, using a password-protected file transfer protocol, the central study PI could view the non-PHI clinical data input by all participating hospitals, without access to PHI from any hospital.

The information required to populate the follow-up e-form required the research coordinator to perform a standardized review of a comprehensive medical record database maintained by the hospital. The first database was a central electronic record storing system where laboratory and radiology results and any transcriptions of dictated clinician notes and optical scanned images could be found for the entire hospital network. This allowed the evaluation of any return visit to the hospital system, (inpatient, ED, clinic or other outpatient visit) to determine if the patient had any of the outcomes of interest in the follow-up form. If no follow-up data were available within the hospital system to prove the patient was alive at 45 days, then the follow-up protocol required query of the public social security master death index to determine if a death certificate had been filed for the patient. Finally, if no valid follow-up were documented by electronic database search, we then attempted to contact the patient through a previously described, stepwise procedure, consisting of a mailed questionnaire, followed by a telephone call, if necessary [7].

When all of the data required for the follow-up form were obtained and input into the form, the research coordinator would complete the form and press the "check form for completeness" button. This would activate a system to ensure that all necessary follow-up data were entered. For example, every patient had to have a valid 45-day follow-up, either in the form of a documented follow-up to a clinic, telephone follow-up with the patient, or confirmation of patient death within 45 days.

## Results

The system was fully implemented on April 15, 2003. As of April 15, 2004, prospective data forms were uploaded from 1022 patients evaluated for acute pulmonary embolism. Prospective data have been entered by 42 clinicians and 6 physician assistants from two hospitals in Charlotte, NC. The primary technical barrier to implementation was the process of loading and using the prospective e-forms on a personal digital assistant. All 48 clinicians required individual help and training, of over one hour each to show them how to install the e-form on their PDAs. This impedance was compounded by real-time difficulties associated with stylus use on a small PDA screen, followed by difficulties with uploading to the website from the PDA led to abandonment of this method of data entry. Out of 48 clinicians, only 6 successfully uploaded more than one e-form from the PDA. Only 12 of 1022 uploaded e-forms originated from a PDA.

The primary technical barrier to implementation on the personal computers included maintaining the URL icon on desktops in the ED (it was occasionally removed by unknown persons). This problem was solved by the permanent link on the hospital's intranet home page. In two separate instances, clinicians reported that they had populated the e-form, attempted to submit, and for unknown reasons, were unable to upload the e-form, and they had to reenter the data and resubmit the e-form. The server has maintained a log of all successive e-forms uploaded by each clinician. No uploaded prospective forms have been lost or deleted. The side-server system was designed to prevent e-form upload with missing or erroneous data.

To examine if this system properly, two observers reviewed the eight parametric field entries (age, heart rate, respiratory rate, systolic blood pressure, pulse oximetry, height, weight and temperature) that were key-entered by clinicians for 1022 patients. Validity required that both observers agree that the entry was a real number within the prespecified range of the parameter. In 44/8176 fields of 12 patients (0.6%), two observers deemed the parametric field entry to be useless for statistical analysis. Stated another way, these data would be coded as missing after data cleaning were completed. However, no categorical data were missing or erroneous. As a result, 1010/1022 (98.8%) of prospective e-forms had usable data in all 70 fields.

Ninety-four percent of all 1022 patients have reported the site hospital to be their hospital of choice. Follow-up forms (Figure 3) have been completed and uploaded on 605 of 1022 patients. Using existing hospital-approved login and authentication procedures, research personnel were able to access necessary databases from their home computers. Thus, using their personal Internet connection and private telephone line, the research associates were able to complete follow-up forms from home. Follow-up forms were completed in an average of 20 ± 12 minutes.

Follow-up has revealed that all prospective e-forms were authentic, and each was completed on an emergency department patient who underwent at least one clinical test for pulmonary embolism. No bogus forms were detected during follow-up to date. This demonstrated a low likelihood of an unauthorized person generating a spoofed form on one of the designated computers in the ED treatment area, given that the automatic control system would not allow a form to be uploaded until all 70 fields are completed.

**Figure 7:**
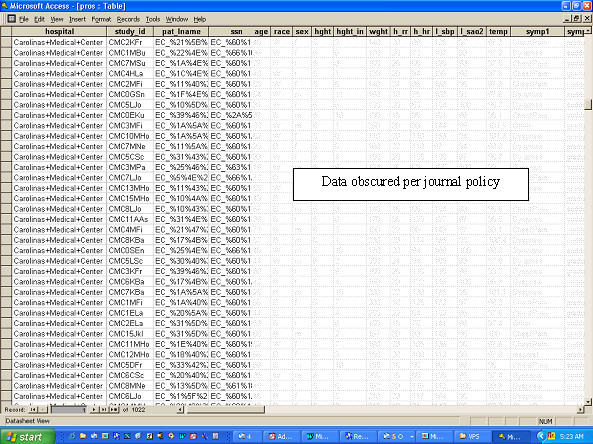
Reproduction of sample data as viewed by the central study PI after file transfer protocol. The non-encrypted patient data, such as age, race and vital signs were obscured by pixellation by the author.

All uploaded prospective and follow-up data were obtained by the central PI using file transfer protocol and were inputted into a spreadsheet without difficulty. Figure 7 shows a screenshot illustrating a partial view of the downloaded study data, including the appearance of the PHI fields after encryption as well as unencrypted data. The purpose of this figure is to demonstrate that the central PI could have access to necessary study data from all sites while remaining blinded to PHI data. The "study ID" field represents the unique identifier used to re-identify PHI data.

Query of the server log revealed no evidence of website hijacking or other intrusion. The server computer which houses the study database and runs the web application uses the Windows^® ^Server 2003 operating system. The only means of electronic access to the server is via hypertext transfer protocol (HTTP) and file transfer protocol (FTP). Both of these system services log all requests made to their ports. An example log entry is shown in the appendix.

## Discussion

The step of obtaining written Authorization to comply with HIPAA can impart a selection bias in registries intended to study acute disease processes [4]. In section 164.512(i), the Privacy Rule allows for waiver of Authorization when the "research could not practicably be conducted without the waiver" and the "use of the PHI involves no more than a minimal risk to the privacy of individuals." The present report tests a system designed to collect clinical data in real-time from patients with acute diseases at multiple hospitals, including a mechanism to facilitate follow-up, while protecting the privacy of the participants.

The first research objective was to determine if PDAs would represent an efficient and secure mechanism for clinicians to record real-time data at the bedside in the emergency department setting. Our experience in this phase I, two-hospital study demonstrated that PDAs presented unexpected complexities that eroded our enthusiasm. The clients were clinicians with variable levels of technical sophistication. Despite our use of a relatively standard process, clinicians found it difficult to download the e-form from the website onto their PDAs, and many needed help from the study authors. Clinicians frequently forgot to bring their PDA devices to work, and during the one-year course of this study, 10 of 48 clinicians bought new PDA devices. Clinicians consistently reported difficulty with the small screen size and data entry with a stylus. Unfortunately, we did not quantify this opinion using a structured survey. We believe this represents the first published experience at using PDAs to collect research data in the emergency department setting. Our results are somewhat less positive than other studies that have reported the use of hand-held computers to maintain clinical databases [8,9]. However, Lu and colleagues previously recognized similar barriers to physician use of PDAs [10]. We emphasize that our protocol was preplanned, adequately budgeted, and technically supported to disseminate the e-form via the PDA. Unfortunately, we did not perform preplanned measurements to explain this failure. We cannot conclude inferiority of the PDA versus other methods (e.g., paper forms or PC platform) for data collection accuracy inasmuch as we did not compare key quality index data (e.g., comparison rate of compliance, missed data, key errors, lost forms) between methods. Thus, we can only explain the failure of the PDA mechanism in the broad terminology of "it lacked feasibility."

The second objective was a relatively complex task intended to determine if the system would allow prospective and follow-up data collection over the Internet in compliance with the requirements of the Privacy Rule. From a functional standpoint, we sought to determine if the system would allow us to protect the data fields that needed to be protected, but allow the non-sensitive data to be accessed by study personnel who did not have local IRB approval. This was accomplished while maintaining strict security standards at each step of data transfer (see Figure 1). Data were uploaded from designated Internet provider addresses via secure socket link protocol and stored in a database on an offsite hosted server that was protected by several layers. No study data could be accessed without a password. Further, the system mandated specific password-protected access to PHI only by IRB- and privacy board-approved individuals at each hospital. This mechanism was designed facilitate the acquisition of patient follow-up data at participating hospitals. However, the central study PI could download the study data of interest via a separate password-protected file transfer protocol, but the PHI data were encrypted (see Figure 7). Because the PHI data were stored on the server after 128 keybit encryption, even in the event of unauthorized data access (hacking), the hijacker would be unable to view the PHI.

Although a large number of commercial systems are available for storing clinical data, most are designed and marketed explicitly for the billing process. In contrast, from the perspective of research, relatively little has been published on the design and implementation of a web-based system to allow collection of clinical data in a multicenter trial design [11,12]. We believe this is the first report of successful web-based clinical data collection under waiver of Authorization and in compliance with CFR 45, parts 160 and 164. This phase I project was limited to two hospitals in the same city, both covered under the same IRB. However, we submit that the system is ready to be expanded to other hospitals in the second phase of the study.

This system was designed to be a reasonably comprehensive tool to obtain key information about the beliefs of a clinician at the time of test ordering. Here, we refer to the clinician's beliefs as what they thought were the values of certain specific clinical data that are commonly used to estimate the pretest probability of pulmonary embolism. To capture these beliefs in real time, the system cannot default to a retrospective review of the patient's chart, or having the clinician complete the form after a shift. Within the emergency department setting, the flow of knowledge is dynamic for each patient. As a consequence of time urgency, emergency clinicians often must decide to order expensive imaging tests based upon limited, changing, and sometimes erroneous information. Occasionally, clinical information becomes updated after an expensive radiological test has been done (e.g., a family member arrives with new information, or medical records arrive from another facility by facsimile). Accordingly, the data collection instrument must accurately capture the information that the clinician uses to motivate his or her test ordering behavior, rather than to collect data after the test results have returned, and more complete medical records may have arrived. 

This report represents a phase I study. For the second phase, we will deploy this system to 10 US hospitals to allow collection of data from 5000 patients. The ultimate goal of this project is to collect a large, multicenter database, as the substrate for a mathematical model to generate a pretest probability of pulmonary embolism based upon beliefs of many clinicians.

## Conclusions

Research data can be successfully collected, entered and uploaded to a hosted server by emergency physicians working in different emergency departments, and in compliance with the Privacy Rule. Use of server side controls to test for data validity ensured that 98.8% of uploaded forms contained complete data usable for statistical analysis. The PHI data were successfully encrypted and deencrypted using password access to allow follow-up at a later date. Server log query demonstrated no evidence of intrusion or data loss, suggesting that data were securely stored.

## Abbreviations

ASP – Active server pages

ED – Emergency department

FTP – File transfer protocol

DHHS – Department of Health and Human Services

HIPAA – Health Insurance Portability and Accountablity Act

HTML – Hypertext markup language

HTTP – Hypertext transfer protocol

IRB – Institutional Review Board

PDA – Personal digital assistant

PHI – Protected health identifiers

PI – Principal investigator

SQL – Standard query language

RUL – Uniform resource locator

## Competing interests

Jeffrey A. Kline is primary author on a US copyright certificate "Electronic Form ("e-form") For Secure Collection of Clinical Data Via the Internet" and inventor on a US patent (pending) that describes the present work. Jeffrey A. Kline and Charles L. Johnson own stock in BreathQuant Medical Systems.

## Appendix

Example of a log entry for the HTTP system service

208.10.156.69 – [02/Mar/2004:23:55:55 +0000] "POST /pestudy/peadmin.asp HTTP/1.1" 302 1349 "" "Mozilla/4.0 (compatible; MSIE 6.0; Windows NT 5.1)"

The IP address of the HTTP requesting browser is **208.10.156.69 **and the resource requested is the ASP file . For the FTP system service this would be a typical log entry

208.10.156.69 – generic [06/Aug/2003:17:00:14 +0000] " [22786]USER generic FTP" 331 0 "-" "-"

The IP address of the HTTP requesting browser is **208.10.156.69 **and the USER identity that was logged in was "**generic"**. Both these log files are scanned once a week to look for any suspicious requests. To date there have been no identified intrusion or hijack attempts on the specific study database. As an HTTP responding server on the public Internet, the web (HTTP) server program does receive many well identified "virus spreading" requests which it denies and whose denials are logged. One such "virus related" request common to all web logs today is

61.100.6.181 – [01/May/2004:11:30:52 +0000] "GET /scripts/nsiislog.dll HTTP/1.1" **404 **3806

This example virus was developed to attack a vulnerability which existed in Microsoft Web Servers but was eliminated by a security update for their web (HTTP) server. The highlighted **404 **code denotes that the request was denied. As security updates from Microsoft become available the study web server is updated. At present there are no known HTTP vulnerabilities which would allow an unauthorized user to gain access to files on the server.

## Authors' contributions

JAK conceived the study, obtained funding and participated in the system design, data collection and drafted the manuscript. CLJ carried out all technical programming, participated in system design and drafting the manuscript. WBW participated in idea conception, system design, data collection and drafting the manuscript. MSR participated in data collection and drafting the manuscript.

All authors read and approved the final manuscript.

## Pre-publication history

The pre-publication history for this paper can be accessed here:



## Figures and Tables

**Table 1:** Dictionary of variables visible to the user via hover buttons

**Hospital of Choice **– The hospital where the patient says he or she receives his or her care. If the patient agrees that the study hospital is his or her hospital of choice, this means that in the patient's opinion, he or she agrees to return to the study hospital for persistent symptoms in the next 45 days.
**Vital signs **– Measured in the ED using local standards and FDA-approved devices. SaO2% must be made with the patient breathing room air or the lowest tolerable rate of oxygen administration.
**Dyspnea **– Patient perception of difficulty breathing at the time of evaluation. Includes sensation described as shortness of breath, breathlessness, labored breathing, trouble breathing, not breathing right.
**Pleuritic Chest Pain **– Focal pain in the thorax located inferior to the transverse axis through the clavicles extending to the costal margin. The pain must change with breathing and must not be reproduced by palpation.
**Substernal Chest Pain **– Pain located behind the sternum that is not reproduced with palpation.
**Chest pain Reproduced with Palpation **– Thoracic pain that increases when palpated gently and the patient agrees that the elicited pain feels similar to the pain that he or she is experiencing.
**Leg or Arm Swelling **– Asymmetry observed on gross inspection. Does not require measurement or presence of edema.
**Wheezing **– Determined by auscultation by any healthcare provider either in prehospital or ED setting.
**Alternative diagnosis **– Another specific disease process for which an ICD-9 code or set of codes exist, including pneumonia, cardiac ischemia, CHF, bronchospasm, muscle strain, acute bronchitis, biliary disease, pneumothorax, aortic dissection, pericarditis, costochondritis. Descriptive diagnoses of atypical chest pain or chest pain of unknown etiology, or Chest pain NOS are not sufficient to be considered alternative diagnosis. Likewise, purely psychiatric diagnoses such as anxiety or fibromyalgia are not to be considered as alternative diagnoses.
**Physician estimate of PTP **– The probability that the patient has PE on the day of evaluation.
**Past Medical History terms**. All terms are based upon the clinician's best estimate of the presence or absence of each condition based upon data available during the ED shift. Data sources may include patient perception, family perception, oral or facsimile communication with other clinicians, evaluation of written or electronic medical records.
**COPD **– In the appropriate clinical setting (eg, history of smoking) a patient affirms yes to the question, "have you been told you have emphysema or damage to your lungs from smoking that has required medical treatment" This diagnosis does require spirometry results.
**Surgery in past 4 weeks**: Includes any surgery requiring general anesthesia.
**Trauma in past 4 weeks**: Any traumatic injury requiring hospitalization for >24 hours or causing fracture requiring stabilization in an ED. Does not include minor laceration repair, scrapes, bruises, concussion, whiplash or soft tissue injuries that did not warrant admission
**Coronary Artery Disease **– Coronary stenoses diagnosed either by clinical grounds if drug therapy is given, or by coronary imaging. Includes prior MI, prior stenting or CABG.
**CHF **– Systolic or diastolic heart failure. Can be based upon clinical diagnosis if drug therapy is given. Does not include diagnosis of CHF made for the first time on the day of enrollment.
**DVT or PE. **Requires that the patient was treated with either caval interruption, or warfarin or fractionated heparin anticoagulation for more than 11 weeks.
**Malignancy **– History of cancer. Decision to term the presence of cancer as "treated and inactive" means the patient perceives that the disease is in remission and the clinician has no evidence to the contrary. If evidence of previously unknown metastases is found in the ED, then the clinician uses the information at hand to code the malignancy status.
**Immobility **– Pathological restriction in body movement. Includes bed-bound patients who cannot or do not walk for periods exceeding 48 hours. Also includes any patient in a cast or with an external fixator in place.
**End stage disease **– Any disease process, such as AIDs, cancer, advanced incurable lung or heart disease with life expectancy < 6 months.
**Follow-up e-form terms**
**Sepsis **– As defined by American College of Chest Physicians
**Discharge Diagnosis **– Based upon written diagnoses and ICD 10 coding
**Troponin **– pull down box allows multiple choices of results for either troponin I or C testing.
**Pressor support **– >5 micrograms of dopamine per kg per min or any use of norepinephrine or epinephrine infusion
